# A comprehensive targeted next‐generation sequencing panel for genetic diagnosis of patients with suspected inherited thrombocytopenia

**DOI:** 10.1002/rth2.12151

**Published:** 2018-10-08

**Authors:** Ben Johnson, Rachel Doak, David Allsup, Emma Astwood, Gillian Evans, Charlotte Grimley, Beki James, Bethan Myers, Simone Stokley, Jecko Thachil, Jonathan Wilde, Mike Williams, Mike Makris, Gillian C. Lowe, Yvonne Wallis, Martina E. Daly, Neil V. Morgan, the UK GAPP Study Group

**Affiliations:** ^1^ Institute of Cardiovascular Sciences College of Medical and Dental Sciences University of Birmingham Birmingham UK; ^2^ West Midlands Regional Genetics Laboratory Birmingham Women's Hospital Birmingham UK; ^3^ Hull York Medical School University of Hull Hull UK; ^4^ Nottingham Haemophilia Centre Nottingham University Hospital Nottingham UK; ^5^ Kent Haemophilia Centre Kent & Canterbury Hospital Canterbury UK; ^6^ Regional Centre for Paediatric Haematology Leeds Children's Hospital Leeds UK; ^7^ Department of Haematology Lincoln County Hospital Lincoln UK; ^8^ Department of Haematology Manchester Royal Infirmary Manchester UK; ^9^ Comprehensive Care Haemophilia Centre University Hospitals NHS Foundation Trust Birmingham UK; ^10^ Department of Haematology Birmingham Children's Hospital Birmingham UK; ^11^ Department of Infection, Immunity and Cardiovascular Science University of Sheffield Medical School University of Sheffield Sheffield UK

**Keywords:** bleeding, gene mutations, targeted panel sequencing, thrombocytopenia

## Abstract

**Background:**

Inherited thrombocytopenias (ITs) are a heterogeneous group of disorders characterized by low platelet counts and often disproportionate bleeding with over 30 genes currently implicated. Previously the UK‐GAPP study using whole exome sequencing (WES) identified a pathogenic variant in 19 of 47 (40%) patients of which 71% had variants in genes known to cause IT.

**Aims:**

To employ a targeted next‐generation sequencing platform to improve efficiency of diagnostic testing and reduce overall costs.

**Methods:**

We have developed an IT‐specific gene panel as a pre‐screen for patients prior to WES using the Agilent SureSelect^QXT^ transposon‐based enrichment system.

**Results:**

Thirty‐one patients were analyzed using the panel‐based sequencing, of which; 10% (3/31) were identified with a classified pathogenic variant, 16% (5/31) were identified with a likely pathogenic variant, 51% (16/31) were identified with variants of unknown significance, and 23% (7/31) were identified with either no variant or a benign variant.

**Discussion and Conclusion:**

Although requiring further clarification of the impact of the genetic variations, the application of an IT‐specific next generation sequencing panel is an viable method of pre‐screening patients for variants in known IT‐causing genes prior to WES. With an added benefit of distinguishing IT from idiopathic thrombocytopenic purpura (ITP) and the potential to identify variants in genes known to have a predisposition to hematological malignancies, it could become a critical step in improving patient clinical management.

**Essentials**

- Inherited thrombocytopenias are a heterogeneous group of disorders with over 30 causative genes identified to date.
- We have developed an IT‐specific gene panel to screen patients using the rapid Agilent SureSelect^QXT^ transposon‐based enrichment system.
- Candidate gene variants were observed in previously implicated IT genes in 77% of individuals; 10% of patients were found to have a classified pathogenic variant, 16% with a likely pathogenic variant, 51% with a variant of unknown significance and 23% with no or benign variant.
- Accurate genetic diagnosis could improve the clinical outcome for this group of patients with disproportionate bleeding for their reduced platelet count.

## 1 Introduction

Inherited thrombocytopenias (ITs) are a heterogeneous group of disorders characterized by a sustained reduction in platelet count often manifesting as a bleeding diathesis. Since the discovery of disease inheritance patterns in disorders such as Bernard Soulier Syndrome (BSS), genetic studies of thrombocytopenia have been a vital tool in determining megakaryocyte and platelet physiology.^1^ As a result of parallel whole exome and whole genome sequencing over the past 5‐10 years, we are discovering increasing numbers of novel genes and variants with a critical role in platelet production, physiology, and function.^2^, ^3^, ^4^, ^5^

To date, there are 30 genes suspected to cause 26 separate forms of inherited thrombocytopenia making genetic diagnosis complex.^6^ However, until recently, IT remained underdiagnosed with previous studies only providing a genetic diagnosis in just over 50% of individuals.^7^, ^8^, ^9^ A genetic diagnosis provides clinical benefits for the patients. Some patients with a reduced platelet count have had unnecessary treatments and procedures such as immunosuppression and splenectomies and therefore establishing that they have an inherited component to their disease etiology would prevent this. In the case of suspected ITP this may be treated with steroids or immunosuppressive drugs with many side effects. Therefore, if such patients are proven to have an inherited thrombocytopenia, then these treatments are unnecessary. Some of the gene mutations in patients, eg, *RUNX1*, result in patients having a predisposition to hematological malignancies and once a genetic defect is proven, the information can be used to monitor the patients’ hematological parameters more closely. These all highlight the need for a definitive genetic diagnosis and development of a targeted gene‐specific sequencing platform will provide a quick and cost effective screening for patients with IT.

As new sequencing library‐capture methods are developed, the speed of sample preparation time is vastly reduced. Thus, the recently released capture methods, Illumina Nextera Rapid Custom Capture Enrichment and Agilent SureSelect^QXT^, both propose an improvement in sample preparation without limitations in sequence depth, coverage, and accuracy.^10^ When applied to small‐scale custom gene panels, the preparation time can be reduced to one day. In addition, DNA input is also reduced allowing for amplification from <50 ng of DNA.^11^

Due to the high percentage of variants within known IT genes as identified by whole exome sequencing (WES) in a previous study,^12^ and the increasing advances in custom panel next generation sequencing, an IT‐specific next‐generation sequencing (NGS) panel was designed and included within the UK‐GAPP patient workflow. Incorporating a small custom panel prior to WES has the potential to filter out variants with a genetic etiology of disease within known IT‐causing genes. Coupled with the Agilent SureSelect^QXT^ transposon‐based system of sample preparation, an increase in the efficiency of genetic diagnosis, as well as a reduction in the overall cost, can potentially be achieved.

Therefore, in this study we aimed to implement a NGS panel in the UK‐GAPP patient workflow. The panel was designed to incorporate all genes known to be previously associated with IT, effectively pre‐screening patients before WES. The targeted panel also takes advantage of a rapid sample preparation technique allowing for quick genetic diagnosis following patient phenotyping and improving overall diagnosis of recruited patients.

## 2 Methods

### 2.1 Patients

Patients were recruited from participating UK hematology centers. All patients had a bleeding history taken at the point of examination and inclusion into the study. Most patients suffered from mild bleeding symptoms including cutaneous bruising, bleeding, and epistaxis in addition to more severe bleeding symptoms in some cases. Detailed patient clinical symptoms related to bleeding that were available are displayed in Table 1.

**Table 1:** Phenotypic symptoms of 31 patients recruited to the UK‐GAPP study with IT of unknown etiology

Patient	Age	Gender	Platelet count (x10^9^/l)	MPV (fL)	IPF (%)	Flow cytometry defect	LTA defect	ATP secretion	Bleeding phenotype
48	5	M	125	9.1	NT	P‐Selectin	NT	NT	Cutaneous bruising, petechiae
49	41	M	30	NA	9.4	NT	ADP	Normal	Cutaneous bruising
50	41	M	30	NA	59.4+	CD42b	NT	NT	Cutaneous bleeding
51	UNK	UNK	162	9.2	NT	P‐Selectin, GPVI	NT	NT	Cutaneous bleeding, oral cavity bleeding
52	43	F	131	8.7	14.7+	Normal	AA	Reduced	Cutaneous bleeding, epistaxis, menorrhagia, Gi bleeding, oral cavity bleeding
53	27	F	104	9.1	NT	Fibrinogen	NT	NT	Cutaneous bleeding
54	12	M	101	10	39.8+	P‐Selectin	NT	NT	Cutaneous bleeding
55	UNK	F	30	8.6	2.3	P‐Selectin	NT	NT	Cutaneous bruising, oral cavity bleeding, menorrhagia
56	15	F	48	10.2	45.2+	Normal	NT	NT	Cutaneous bleeding, epistaxis
57	11	F	153	12.1	9.2	NT	ADP, AA	Reduced	Cutaneous bruising/bleeding
58	9	F	82	8.6	6.4	P‐Selectin, Fibrinogen	NT	NT	Cutaneous bruising/bleeding
59	4	M	94	12.3	7.1	Normal	NT	NT	Cutaneous bruising/bleeding
60	UNK	F	146	13.4+	15.8+	CD41	Adr	Normal	Cutaneous bruising, oral cavity bleeding, menorrhagia
61	UNK	F	76	9.7	3.4	P‐Selectin, Fibrinogen	NT	NT	No observable phenotype
62	34	F	138	13.8+	17.5+	Normal	NT	NT	Cutaneous bleeding
63	13	F	37	14.6+	16+	P‐Selectin	NT	NT	Cutaneous bruising, petechiae, epistaxis
64	UNK	F	105	14.5+	23.1+	NT	Normal	Normal	Cutaneous bruising, epistaxis
65	35	F	52	14.9+	19.4+	P‐Selectin, Fibrinogen	NT	NT	Cutaneous bruising/bleeding
66	18	F	87	10	1.8	P‐Selectin, Fibrinogen	NT	NT	Cutaneous bruising/bleeding
67	22	M	40	13.1+	15.7+	NT	Normal	Normal	Cutaneous bruising/bleeding, epistaxis
68	17	M	191	NT	NT	NT	NT	NT	Cutaneous bleeding, nose bleeds
69	26	M	69	NT	NT	NT	NT	NT	Nose bleeds
70	34	F	96	NT	NT	NT	NT	NT	None, incidentally identified thrombocytopenia
71	50	M	128	NT	NT	NT	NT	NT	None, investigated as son has thrombocytopenia
72	33	F	14	NT	NT	NT	NT	NT	Cutaneous bleeding, menorrhagia, acute lymphoblastic leukemia, father died of acute myeloid leukemia
73	29	F	53	11.7	9.1	NT	NT	NT	Cutaneous bruising, hematuria, oral cavity bleeding
74	72	M	50	8.4	NT	P‐Selectin	NT	NT	Cutaneous bruising, epistaxis
75	48	F	92	NT	NT	NT	NT	NT	Cutaneous bruising, hematomas
76	UNK	F	92	10.2	NT	Fibrinogen	NT	NT	No observable phenotype
77	15	F	76	9.4	13.5+	Normal	NT	NT	Oral cavity bleeding, menorrhagia
78	UNK	M	101	13.9+	14.4+	Normal	Adr	Normal	Cutaneous bleeding

Average platelet count = 88 × 10^9^/L (normal range to 2 SD 147‐327 × 10^9^/L, n = 40). Average MPV = 11.1 fL (mean normal range to 2 SD 7.8‐12.69 fL, n = 40). IPF was available for 20 patients and varied between 1.8‐59.4% (normal range 1.3‐10.8%, n = 40). Patients with an observed macro and micro thrombocytopenia are denoted by a + and ‐, respectively, following their most recent analyzed MPV. Secondary qualitative defects are abbreviated to the following; (CD41) reduction in the resting cell surface levels of CD41, (CD42b) reduction in resting cell surface levels of CD42b, (ADP) reduction in response upon ADP stimulation indicating a possible defect in the Gi pathway, (AA) reduction (cyclooxygenase pathway defect), (Adr) reduction (Thromboxane receptor pathway defect), (GPVI) reduction in surface GPVI quantity, (P‐selectin) reduction (platelet alpha‐granule/secretion defect), (fibrinogen) reduction in the binding of fibrinogen to activated platelets, (ATP secretion) reduction in ATP secretion upon stimulation with PAR‐1 peptide 100 μmol/L. Bleeding diathesis of each individual is summarized under bleeding phenotype.

AA, arachadonic acid; ADP, Adenosine diphosphate; Adr, adrenaline; ATP, Adenosine triphosphate; GPVI, Glycoprotein VI; IPF, immature platelet fraction; LTA, light transmission aggregometry; MPV, mean platelet volume. + denotes an elevated MPV/IPF; NA indicates parameter was tested but results were inconclusive; NT indicates parameter was not tested due to degraded or limited sample; UNK indicates the parameter was not known.

The UK‐GAPP study was approved by the National Research Ethics Service Committee of West Midlands—Edgbaston (06/MRE07/36) and participants gave written informed consent in accordance with the Declaration of Helsinki. The GAPP study was registered at http://www.isrctn.org as #ISRCTN 77951167 and is included in the National Institute of Health Research Non‐Malignant Haematology study portfolio, (ID‐9858).

### 2.2 Platelet counts and morphology

Platelet counts and morphology were measured from patients in whole blood using the Sysmex XN‐1000 (n = 31). The PLT‐F channel was used to determine platelet counts in whole blood and the immature platelet fraction (IPF). Mean platelet volume (MPV) was determined from the impedance PLT‐I channel. All samples were processed in tandem with travel controls.

### 2.3 Platelet preparation and platelet function testing

This study focuses on a subset of patients with a reduction in platelet count. Previous studies by the UK‐GAPP study group have demonstrated the applicability of using light transmission aggregometry (LTA), including lumiaggregometry, for investigation of PRP samples having platelet counts exceeding 1 × 10^8^/mL^13^ and an in‐house flow‐cytometry assay to assess platelet function in patients having platelet counts in PRP of less than 1 × 10^8^/mL.^12^

### 2.4 Thrombocytopenia‐specific panel sequencing

A thrombocytopenia panel was designed for use as an initial NGS (NGS) sequencing/pre‐screen before whole exome sequencing in collaboration with the Regional Genetics laboratory at Birmingham Women's Hospital.

The panel was designed using the Agilent SureDesign v3.5.4 (Agilent Technologies, UK) design software. The original design included the following 30 genes; *ABCG5*,* ABCG8*,* ADAMTS13*,* ANKRD18A*,* ANKRD26*,* CYCS*,* FLI1*,* FLNA*,* FYB*,* GATA1*,* GFI1B*,* GP1BA*,* GP1BB*,* GP5*,* GP9*,* HOXA11*,* ITGA2B*,* ITGB3*,* MKL1*,* MPL*,* MYH10*,* MYH9*,* NBEAL2*,* ORAI1*,* RBM8A*,* RUNX1*,* SLFN14*,* STIM1*,* TUBB1*, and *WAS*. The 30 genes encompassed genes previously associated with IT as well as some of their related genes and novel genes identified as being associated with thrombocytopenia as part of the GAPP study. This panel was applied to patients 48 to 61 (inclusive) and patients 72 and 73, which encompass the first 16 patients that were analyzed by panel sequencing. Sequencing probes/baits were designed to cover the following regions: all coding exons ±10 bp flanking sequence from the intron‐exon boundary and the 5′UTR and 3′UTR. Sequencing baits were designed with 2x density so that each desired region was covered by at least two overlapping probes. Baits were also designed with the strictest masking stringency settings possible. SureDesign masks repetitive sequences dependent on three masking tools: RepeatMasker, WindowMasker, and Uniqueness 35 track. The design software uses combinations of all three tools to create three masking stringencies which vary in their inclusiveness of repeat regions. If baits could not be found in the highest stringency possible, stringency was decreased until they could be found. Eighteen genes were covered entirely using the highest stringency setting, eight genes were covered by a combination of high and moderate stringency settings, and the remaining four genes were covered by baits using a combination of all three stringency settings. Balanced boosting of GC‐rich probes was used which replicated the amount of probes within a GC‐rich region by a defined factor to improve capture of these difficult genomic fragments. The final design incorporated 3309 probes with an overall size of 212.189 kbp.

**Figure 1:**
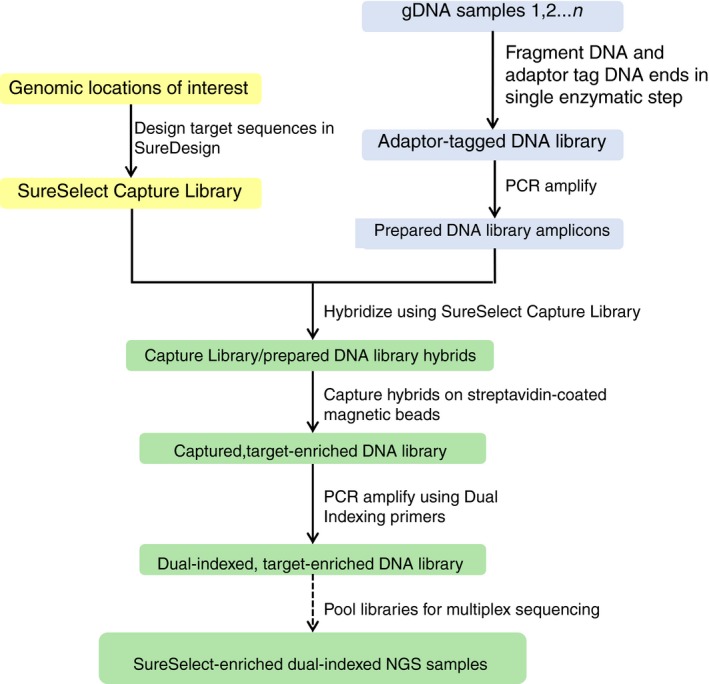
Sample preparation workflow for the IT‐specific next‐generation sequencing panel using Agilent SureSelectQXT capture methodology

For subsequent sequencing beyond the first group of 16 patients as detailed above, an improved design was utilized to include new genes implicated in IT. The second version of the design included all probes from the first design with the addition of baits designed to sequence the following genes; *ACTN1*,* ETV6*,* PF4*, and *PRKACG*. All genes, with the exception of *PRKACG*, were covered by probes with the most stringent masking settings. Probes designed for amplification and sequencing of *PRKACG* included four probes with the least‐stringent masking settings applied. Overall the new design incorporated 3447 probes covering 221.305 kbp. Target enrichment was performed for all designs using the Agilent SureSelect^QXT^ NGS target enrichment kit for Illumina multiplexed sequencing (Agilent Technologies). Sample preparation followed the workflow outlined in the manufacturer's instructions (Figure 1). Due to the relative small size of the capture library all size‐related steps followed the methodology for capture libraries <3 Mb. In the preparation for hybridization 750 ng of gDNA, diluted in a volume of 12 μL was used.

A maximum of 16 samples were prepared per run. DNA samples were quantified using BR and High Sensitivity (HS) Qubit dsDNA fluorometric quantification kits and were analyzed using a Qubit 2.0 Fluorimeter (ThermoFisher, UK, #Q32854 for HS kit) in initial sample preparation. Purification steps utilized Agencourt AMPure XP magnetic capture beads (BeckmanCoulter, UK, #A63880). DNA quantity and quality was assessed at two separate points throughout the protocol using an Agilent 2200 Tapestation system and associated D1000 and high sensitivity D1000 screen tape (#5067‐5582 for D1000, #5067‐5584 for HS D1000), and reagents (including ladder and sample buffers) (#5067‐5583 for D1000 reagents, #5067‐5585 for HS D1000 reagents) (Agilent Technologies). Dynabeads MyOne Streptavidin T1 magnetic beads were used for hybrid capture (ThermoFisher, #65601). Index tags were added using the SureSelect^QXT^ P7 and P5 dual indexing primers. All thermocycling steps were performed using a Bio‐Rad DNA Engine Tetrad 2 Thermal Cycler (Bio‐Rad, UK). Magnetic separation was achieved using a DynaMag‐96 Side magnet (ThermoFisher).

Samples were then pooled for multiplexed sequencing so that each index‐tagged sample was in equimolar amounts in the pool. For each sample the following formula was used to determine the amount of index sample to use.

$$\text{Volume of Index}=\frac{V(f)\times C(f)}{\#\times C(i)}$$

where *V* (f) = Final desired volume of pool; *C* (f) = Desired final concentration of all DNA in pool; # = is the number of the indexes; *C* (i) = Initial concentration of each sample.

A final desired volume of pool of 20 μL was used and a final concentration of 4 nmol/L. In all cases 16 indexes were pooled.

To achieve an optimal cluster density, a final concentration of 10‐pmol/L DNA was used. DNA was firstly denatured by the addition of 5 μL of 0.2 mol/L NaOH to 5 μL of 4 nmol/L pooled library and allowed to incubate at room temperature (~20°C) for 5 minutes before adding 990 μL of pre‐chilled HT1 hybridization buffer was added to achieve a 20‐pmol/L solution. A 300‐μL aliquot of the 20‐pmol/L solution was diluted with 300 μL HT1 to achieve a final concentration of 10 pmol/L in 600 μL.

Finally sequencing was performed using an Illumina MiSeq (Illumina, UK) and MiSeq v2 300 Cycle Reagent Kits (Illumina, #15033626). Sample sheets were designed to allow for the use of custom primers and no adaptor trimming. Sequencing followed a Nextera XT sample preparation kit and amplicon chemistry.

Sequence alignment, annotation, categorisation and variant calling was performed using the SureCall v3.5 software (Agilent Technologies) and the GenAligners 3.0 alignment tool (Agilent Technologies). Post aligned, annotated, and categorized sequence data was analyzed using a personalized bioinformatics pipeline as discussed below.

### 2.5 Bioinformatics pipeline to determine candidate variants

Sequence data generated using the IT‐specific NGS panel was analyzed using an adaptation of the pipeline developed for the analysis of WES data.^12^ Variants were initially filtered on frequency, excluding variants with a MAF ≤0.01 in the 1000‐G database. Synonymous variants not predicted to change the amino acid sequence in the protein coding transcripts were then excluded. As the panel was designed to include the 5′ and 3′ UTRs, variants were additionally filtered dependent on their genomic location within the coding region ±10 bp of intron‐exon boundaries. The UTRs were included in bait design to allow detection of variants within the 5′ UTR of *ANKRD26* so that all variants occurring within the 5′UTR of genes were analyzed individually. Candidate variants identified were scrutinized using the same in silico pathogenicity prediction software and variant classification system as candidates from WES analysis as outlined previously.^12^ Finally, pathogenicity of variants was determined and called using the consensus guidelines as set out by the American College of Medical Genetics and Genomics and the Association for Molecular Pathology (hence forth referred to as the ACMG guidelines).^14^

### 2.6 Quality of sequence data, average number of variants, and sequence coverage

All individual DNA samples were processed and passed QC at two points during sample preparation. Prior to sample pooling an average calibrated DNA concentration of 2.957 ng/μL and a molarity of 13.3 nmol/L was observed across all samples. All sequencing runs passed internal QC that is used within the West Midlands Regional Genetics Service at the Birmingham Women's Hospital and internal QC from the SureCall analysis software. All candidate variants identified were classified as high quality mapped variants with a quality score of 255 using the SureCall software.

## 3 Results

### 3.1 Phenotyping of IT patient cohort recruited to the study

All 31 unrelated cases included in this study underwent clinical evaluation to exclude idiopathic thrombocytopenic purpura (ITP) (following a relatively stable reduced platelet count over time) and other nonplatelet disorders including von Willebrand disease and inherited coagulation factor deficiencies. Subsequent analysis after enrolment isolated this group of patients with a suspected platelet count less than 150 × 10^9^/L and therefore suspected to have an inherited thrombocytopenia of unknown etiology (Table 1). Platelet counts varied between 30 and 162 × 10^9^/L among the 31 individuals with a mean count of 88 × 10^9^/L (Table 1) (normal range to two standard deviations 147‐327 × 10^9^/L, n = 40). Patients with a platelet count between 150‐200 × 10^9^/L were retained for analysis within the study under the stipulation that there was a shared phenotype within the patient and related affected family members and that a prior platelet count has been below 150 × 10^9^/L. Mean platelet volumes were between 8.6‐14.6 fL (n = 23) (mean for healthy controls ±2 SD = 7.8‐12.4 fL).The immature platelet fraction (IPF) mean in the patients was 17% of the total platelet count (range 1.8‐59.4%), n = 20 (normal range 1.3‐10.8%, n = 40, mean 4.4%),the higher values reflecting abnormal bone marrow platelet production or thrombopoiesis.

LTA was used to assess platelet function for seven patients and flow cytometry alone was performed for samples from 17 patients. Platelets from individuals with a borderline platelet count in PRP of between 0.1 and 0.15 × 10^9^/L were assessed using both assays (n = 3). Platelet function studies revealed the suggestion of the presence of a secondary qualitative defect in addition to the reduction in platelet count in 19 of 24 (79%) (Table 1) of the overall cohort of patients tested.

### 3.2 IT‐specific NGS panel

On average, 326 variants were noted in samples from each individual analyzed by the IT‐specific NGS panel. This ranged from 265 to 400 variants across all samples analyzed. Following exclusion of synonymous variants, an average of 73 variants, having a MAF ≤0.01 in the 1000‐G database were noted per individual. When the ExAC database was interrogated for each of the variants identified in non UTR regions only those variants displayed in Table 3 were of a rare frequency (MAF <0.01).

Average coverage across the targeted regions was in excess of 95% for all samples analyzed. An average read depth of 380 was noted at the site of each variation. This read depth was not observed below 121 at each point of all candidate variants and reached a filtered read depth of 823.

### 3.3 Validation of IT‐specific NGS panel

Validation of the IT‐specific NGS panel was performed by analyzing the panel's sensitivity in detecting eight variants identified previously by WES analysis and confirmed by Sanger sequencing. Variants, at the time of validation, were likely candidate variants and include variants in genes not known previously to cause IT (see Table 2). All variants, excluding a previously identified frameshift causing insertion in *TUBB1*; c.1080_1081insG, p.Leu361Alafs*19 previously identified using WES in patient 31,^12^ were successfully identified, presumably due to the sequence context around this genomic region. All known candidate variants tested were the only candidate variants following bioinformatics analysis of panel sequencing results in each patient.

**Table 2:** Eight patients and the eight known candidate variants^12^ representing a range of mutation types utilized for the validation of the IT‐specific next‐generation sequencing panel

Patient	Gene	Variation	Type
2	*ANKRD26*	c.‐126T>G	5′‐UTR
17	*RUNX1*	c.G236A, pTrp79*	Nonsense
20	*RUNX1*	c.G332A, p.Gly108Ser	Partial heterozygous missense
21	*RUNX1*	c.427 + 1G>T	Splice site variant
26	*SLFN14*	c.A652G, p.Lys218Glu	Missense
31	*TUBB1*	c.1080_1081insG, p.Leu361Alafs*19	Frameshift causing insertion
36	*WAS*	c.G1456A, p.Glu486Lys	X‐linked Missense
41	*ANKRD18A*	c.2395_2397del, p.Glu799del	Non‐frameshift causing deletion

### 3.4 Candidate variants observed and variant prevalence in 31 new patients

In total, DNA samples from 31 new patients were analyzed by an IT‐specific NGS panel. All patients, with the exception of 64, were single affected cases. Patient 64 forms part of a pedigree of four affected family members which will be discussed in more detail in the discussion section. Following post‐sequencing bioinformatics analysis candidate variants previously implicated in IT genes were observed in 77% of individuals (Table 3). In total, 37 variants were noted in the 24 patients observed with a genetic variant in a gene previously known to cause IT. Seven patients were observed with two variants in two different genes and three patients were noted to have three variants. No patients were noted with two variants occurring within the same gene and all variants were observed in a heterozygous state. One variant; *GP5*; c.867G>C, p.Met289Ile, was noted in two unrelated patients, 57 and 58.

**Table 3:** Variants identified by analysis of the IT‐specific next‐generation sequencing pane

Patient	Gene(s)	Genomic variation	Protein effect	Variation type	Prevalence	PhyloP
48	*ABCG5*	c.293C>G	p.Ala98Gly	Missense	0.005 (rs145154937)	5.277
	*ABCG8*	c.1667T>C	p.Phe556Ser	Missense	0.0009 (rs548098742)	2.914
	*TUBB1*	c.‐88G>C		5′UTR start gain	0.004 (1000G) (rs150072434)	
49	*ABCG5*	c.1864A>G	p.Met622Val	Missense	0.00541 (rs140374206)	−0.748
	*NBEAL2*	c.6631G>A	p.Asp2211Asn	Missense	Novel	5.515
50	*CYCS*	c.155C>T	p.Ala52Val	Missense	Novel	5.962
	*GP1BB*	c.del120‐142	p.Arg42Cys fs*14	Frameshift deletion	Novel	
51	*FLI1*	c.812G>A	p.Arg271Gln	Missense	Novel	5.983
	*MYH9*	c.2872G>A	p.Ala958Thr	Missense	0.0009 (rs151036570)	6.088
52	*FLNA*	c.5948C>T	p.Ser1983Leu	Missense	0.0026 (rs187029309)	5.952
53	*FLNA*	c.7583A>T	p.Asp2528Val	Missense	Novel	4.858
	*MYH9*	c.7C>G	p.Gln3Glu	Missense	0.0015 (rs56200894)	4.643
	*TUBB1*	c.1199G>A	p.Ser400Asn	Missense	Novel	5.88
54	*GATA1*	c.1240T>C	p.*414Arg+41	Stop loss	Known	2.408
55	*GP1BA*	c.206C>T	p.Pro69Leu	Missense	0.001872 (rs138825640)	−1.407
56	*GP1BB*	c.242T>G	p.Leu81Arg	Missense	Novel	−0.162
57	*GP5*	c.867G>C	p.Met289Ile	Missense	0.003101 (rs142440028)	2.516
58	*GP5*	c.867G>C	p.Met289Ile	Missense	0.003101 (rs142440028)	2.516
	*STIM1*	c.182A>G	p.Glu61Gly	Missense	0.00004941 (rs202160755)	2.851
59	*ITGA2B*	c.886G>A	p.Gly296Arg	Missense	Novel	3.205
	*RUNX1*	c.386C>A	p.Ala129Glu	Missense	Known (rs267607026)	6.077
60	*ITGA2B*	c.2176A>T	p.Lys726*	Nonsense	Novel	1.419
61	*ITGA2B*	c.2417G>A	p.Ser806Asn	Missense	Novel	0.148
	*WAS*	c.995T>C	p.Val332Ala	Missense	0.0051 (rs2737799)	0.096
62	*MKL1*	c.569C>T	p.Pro190Leu	Missense	0.00016 (rs200309955)	3.693
63	*MKL1*	c.1492G>C	p.Val498Leu	Missense	0.000008638 (rs199750225)	2.138
64	*MYH9*	c.2152C>T	p.Arg718Trp	Missense	Known	2.044
65	*MYH9*	c.5074G>A	p.Ala1692Thr	Missense	Novel	4.087
66	*MYH10*	c.2987C>T	p.Ala965Val	Missense	0.0079	4.822
	*NBEAL2*	c.4361C>T	p.Thr1454Met	Missense	0.0001	3.227
67	*TUBB1*	c.421G>A	p.Gly141Arg	Missense	0.00003295 (rs778975827)	5.803
68	*ABCG8*	c.1629G>T	p.Arg543Ser	Missense	0.0002 (rs201690654)	5.057
69	*ACTN1*	c.136C>T	p.Arg46Trp	Missense	0.00000827	5.532
70	*GFI1B*	c.503G>T	p.Cys168Phe	Missense	0.0006011 (rs527297896)	4.334
71	*RUNX1*	c.86T>C	p.Leu29Ser	Missense	0.01629 (rs111527738)	0.683
	*NBEAL2*	c.4085G>A	p.Arg1362Gln	Missense	0.00000829	1.666
	*GFI1B*	c.551G>C	p.Arg184Pro	Missense	0.00000746	1.433
72	Unknown					
73	Unknown					
74	Unknown					
75	Unknown					
76	Unknown					
77	Unknown					
78	Unknown					

Patient	Phastcons	Mutation taster	PolyPhen‐2	SIFT	Provean	ACMG criteria	Classification
48	1	D	D	D	D	PP3	Uncertain significance
	1	D	D	D	D	PP3	Uncertain significance
							Uncertain significance
49	0	P	B	T	N	BP4	Uncertain significance
	0.997	D	D	D	D	PM2, PP3	Uncertain significance
50	1	D	B	D	D	PM2	Uncertain significance
		D				PM2, PP3, PM4, PP4	Likely pathogenic
51	1	D	D	D	D	PM2, PP3	Uncertain significance
	1	D	B	T	N		Uncertain significance
52	1	P	D	D	D		Uncertain significance
53	1	D	D	D	D	PM2, PP3,	Uncertain significance
	0.998	D	B	D	N		Uncertain significance
	0.972	D	B	D	N	PM2	Uncertain significance
54	0.572	P				PM4, PS1, PP5	Likely pathogenic
55	0	P	B	D	D		Uncertain significance
56	0.175	P	D	D	D	PM2, PP4	Uncertain significance
57	0.55	P	B	D	D		Uncertain significance
58	0.55	P	B	D	D		Uncertain significance
	1	D	B	D	D		Uncertain significance
59	0.999	D	B	D	D	PM2	Uncertain significance
	1	D	D	D	D	PP3, PS1, PS3	Pathogenic
60	0.957	D				PM2, PM4, PVS1, PP4	Pathogenic
61	0.286	P	B	T	N	PM2	Uncertain significance
	0	P	B	T	N	BP4	Uncertain significance
62	0.987	D	B	T	D		Uncertain significance
63	0.998	D	B	T	N		Uncertain significance
64	1	D	D	D	D	PP3, PS1, PS3, PM (segregation), PP4	Pathogenic
65	1	D	B	T	N	PM2	Uncertain significance
66	1	D	B	D	D		Uncertain significance
	0.999	D	B	T	N		Uncertain significance
67	1	D	D	D	D	PP3	Uncertain significance
68	1	D	D	D	N	PM2	Uncertain significance
69	1	D	D	D	D	PS1, PM2, PP3	Likely pathogenic
70	1	D	D	D	D	PS1, PM2, PP3	Likely pathogenic
71	1	P	D	T	N		Uncertain significance
	1	P	D	T	N		Uncertain significance
	0.995	D	D	D	D	PS1, PM2, PP3	Likely pathogenic
72							
73							
74							
75							
76							
77							
78							

Prevalence is shown in the ExAC consortium if not specified otherwise. PhyloP and Phastcons scores are shown. Variants are noted as D, disease causing and P, polymorphism in MutationTaster; D, damaging and T, tolerated in SIFT; D, deleterious and N, neutral in Provean; D, damaging and B, benign in PolyPhen‐2 in silico pathogenicity prediction software. PhyloP scores vary between −14 and +6 and measure conservation at each individual base, sites predicted to be conserved are assigned a positive score, fast‐evolving sites are assigned a negative score. Mutationtaster uses a Beyes classifier to predict the effect of a mutation from a feed a classifiers. SIFT damaging prediction score = <0.05. Provean deleterious score = < −2.5. PolyPhen‐2 predictions are appraised qualitatively as benign or damaging. The ACMG consensus guidelines, including supporting evidence, are also shown.

The majority of variants identified were missense variants affecting a single amino acid. This equated to 89% of the variants observed. In addition; one 5′UTR start gain was noted in patient 48 (*TUBB1*; c.‐88G>C), one frameshift causing deletion was noted in patient 50 (*GP1BB*; c.del120‐142, p.Arg42Cys fs*14), one stop loss variant was noted in patient 54 (*GATA1*; c.1240T>C, p.*414Arg+41), and one nonsense causing SNV was observed in patient 60 (*ITGA2B*; c.2176A>T, p.Lys726*).

Of the 37 variants, 11 (30%) were novel and not previously identified in any of the databases scrutinized. Twenty‐six variants have been observed previously and the prevalence of these variants in the ExAC database, unless otherwise stated, is displayed in Table 3. When comparing all previously observed variants an average MAF of 0.00256 is noted. All variants were observed at a frequency of less than 0.01 and all previously identified variants, with the exception of rs111527738 which was present within the latest build of dbSNP. Four pathogenic or likely pathogenic variants were identified that are previously known to cause IT. These were found in patients; 54 (*GATA1*; c.1240T>C, p.*414Arg+41), 59 (*RUNX1*; c.386C>A, p.Ala129Glu), 70 (*GFI1B*; c.503G>T, p.Cys168Phe), and 64 (*MYH9*; c.2152C>T, p.Arg718Trp).^3^, ^15^, ^16^, ^17^

Patient 54, a 12‐year‐old male with a history of cutaneous bleeding and a mild reduction in platelet count (101 × 10^9^/L) was noted with a stop loss variant in *GATA1*; c.1240T>C, p.*414Arg+41. The predicted effect of variation is a loss of the wild type stop codon and extension of the protein by 41 amino acids. Most reported variants within *GATA1* occur within the N‐terminal zinc finger domain, leading to a disruption of the binding of GATA1 to FOG1. The stop‐loss variant noted in patient 54, was first identified in a 67‐year‐old male proband who suffers from easy bruising.^16^ The patient's platelet counts varied between 86 to 94 × 10^9^/L at different times of testing and no other differences in hematological cell numbers were noted. The patient was initially sequenced due to the presence of a rare X‐linked blood group Lu(a‐b‐) phenotype which results in the marked decrease in expression of Lutheran glycoprotein on the erythrocyte cell surface. To date, serological analysis using flow cytometry to analyze the presence of Lutheran on the erythrocyte cell surface has not been undertaken in patient 54. Also the presence of giant occasional macrothrombocytes, a marker of the published phenotype, have not been observed in patient 54 in routine histological examination.

A previously identified causative variant was noted in *RUNX1* in patient 59. The missense variant, c.386C>A, p.Ala129Glu, was found in addition to a missense variant in *ITGA2B*. The variant has previously been reported to be causative of FPD/AML in three patients from a single pedigree.^15^ All three patients were identified with the p.Ala129Glu germline mutation causative of FPD/AML. All patients developed AML as a result of a secondary somatic event occurring within *RUNX1* progressing to patient death in all cases. Patient 59 is a male with a mild reduction in platelet count to 94 × 10^9^/L. Following platelet function testing no reduction in platelet secretion (a hallmark of variants within *RUNX1*) was noted. However, it is highly likely that the variant observed in *RUNX1* is causative of the hemostatic phenotype observed. Whether the variant within *ITGA2B* is additive to the phenotype is unlikely as the platelet count is considered mild in severity but may warrant further investigation.

Patient 64, is the only patient analyzed by the IT‐specific panel for whom affected family members were also recruited to the study. The patient forms part of a pedigree of four affected family members with a shared phenotype and clinical symptoms. Following analysis of the IT‐specific panel sequencing, a missense variant was identified in *MYH9*; c.2152C>T, p.Arg718Trp. This variant has been noted once previously in a patient initially diagnosed with MYH9‐RD.^3^ The variant occurs within the motor domain of MYH9 and is associated with an increased risk of deafness and nephritis, however, no secondary symptoms have previously been reported in patient 64 or any of the affected family members also recruited to the UK‐GAPP study. However patients such as this should be monitored regularly for signs of kidney disease. Two variants previously identified by WES analysis of 69 patients were also identified in patients analyzed by the IT‐specific panel sequencing. These variants; *CYCS*; c.155C>T, p.Ala52Val, and *ITGA2B*; c.2176A>T, p.Lys726* were identified in patients 50 and 60, respectively.

### 3.5 Conservation, pathogenicity prediction, and variant classification

Conservation at the site of variation was determined by PhyloP and PhastCons in silico software. Conservation scores for all variants occurring within known IT‐causing genes in the 31 patients are shown in Table 3. Average scores of 3.32887 and 0.829571 were observed across all variants in PhyloP and PhastCon analysis, respectively. The majority of variants occurred at sites of high conservation and the two methodologies used were in agreement in all instances.

Pathogenicity was predicted using in silico prediction software as displayed in Table 3. Classification often varied amongst the software used for each variant indicating the benign potential of the variants observed.

In total, of the 37 total variants noted across all patients investigated, three variants were classified as “pathogenic” and five “likely pathogenic” when considering the ACMG consensus guidelines. The remaining 29 variants without a positive prediction of pathogenicity were classified as of “unknown significance.” Only two variants displayed supporting evidence for a benign classification. Of the 24 patients where a genetic variant was identified this classification predicted equated to 12% (3 of 24) of patients with a pathogenic variant, 21% (5 of 24) with a likely pathogenic variant, and 67% (16 of 24) variants of unknown significance.

## 4 Discussion

An IT gene–specific NGS panel was developed in order to pre‐screen patients prior to WES. The aim was to filter out patients with variants in known IT‐causing genes allowing subsequent focus on WES for patients who may harbor variants in novel genes. In addition, the cost implications were an important consideration given that the WES was more than four times as expensive compared with targeted panel sequencing.

All sequencing passed QC at all points throughout sample preparation and QC, cluster density and overall sequencing data was sufficient when compared with routine sequencing using alternate capture methods performed. Although considered a rapid capture method, Agilent SureSelect^QXT^ sample preparation does not quite reach optimum depth of coverage, evenness and target enrichment when compared with alternate methods of capture including Agilent SureSelect^XT^.^10^, ^18^ When applied to our custom designed panel, average coverage easily exceeded a universally accepted minimum 20x coverage for efficiently calling variants and an average read depth of 380 was identified at the points of variation.^19^

With a GC content of 73%, *GP1BB* often suffers from a reduction in coverage, which is why in WES analysis the gene was manually analyzed. Utilizing the NGS panel there was no drop in coverage within *GP1BB* for all patients analyzed and two variants, in patients 50 and 56, were identified, which may be causative of disease. This represents variants which could be potentially missed by other sequencing methodologies and potential advantage of panel‐based sequencing.

In total, candidate variants, that could be considered for further analysis, were identified in 77% of individuals when analyzed by the IT‐specific panel. This detection rate is in keeping with other recent previous large‐scale targeted panel sequencing studies and the application of WES to patients with IT of unknown etiology.^4^, ^12^, ^20^, ^21^ One possible explanation for the inflated detection rate for panel based platforms is the relative increase in average read coverage when compared to WES analysis, especially at the point of variation.

When comparing prevalence, however, next‐generation panel sequencing identifies a large number of variants that have been previously identified with a low MAF. This may be an indication that the variants are tolerated within the population and are not causative of disease. One way to determine this would be to analyze the co‐segregation of variants within affected/unaffected relatives of the index cases. This has the potential to rule out or further strengthen any identified variants but unfortunately in this study this information was unavailable. The most comprehensive database of genetic variation is noted to be the ExAC database,^22^ which includes data from the aggregation and analysis of high‐quality exome sequence data for 60 706 individuals of diverse ancestries generated as part of the Exome Aggregation Consortium (ExAC). It is plausible, therefore, that although the variants have previously been noted, they are causative of a mild reduction in platelet count that has, or has not, been previously diagnosed in all other patients with the shared variant. To determine the reality of this would require further conformational work.

Comparing pathogenicity prediction and variant classification to the variants determined by WES analysis, a larger percentage of variants were deemed to be of unknown significance. This may reflect a reduced rate of sensitivity and a higher proportion of false negative variants identified. However, it is worth considering that the majority of variants, 62% (23 of 37), displayed supporting evidence of pathogenicity but lacked sufficient evidence to be classified as such. This could potentially be an indication of the lack of strengthening evidence that is normally provided in the form of related affected family members that would be recruited to the study, negatively affecting classification because of a lack of segregation analysis.

The presence of a variant in *MYH9* in patient 64 highlights the difficulty of picking up such defects despite the routine pre‐screening for disorders such as BSS‐ and MYH9‐related disease (using flow cytometry or the presence of granulocyte inclusions, respectively) in hemophilia care centers before recruitment to the UK‐GAPP study. This was also the case in our previous study where we employed WES and detected MYH9 and BSS defects despite pre‐screening by the referring laboratories.^12^ Analyzing patients using the IT‐specific panel has elucidated variants in genes known to cause BSS‐ and MYH9‐related disease in seven patients. With the exception of patients 50 and 65, who present with the characteristic increase in MPV to the magnitude of observable giant platelets, the remaining patients show an unaltered MPV. No Döhle‐like body leukocyte inclusions were noted on peripheral blood smears of patients 64 and 65 and no patients presented with secondary symptoms relating to specific IT disorders. However it should be noted that not all MYH9 defects are associated with the presence of Dohle‐like bodies in a peripheral blood smear. The defects identified may therefore be causative of non‐typical forms of BSS‐ and MYH9‐related IT but in order to exclude a MYH9 defect conclusively, immunofluorescence should be performed for the non‐muscle myosin heavy chain protein.

A phenotype‐genotype correlation is often utilized in aiding in the diagnosis of a patients disease. Patient 61 presented with a marked reduction in the cell surface levels of CD41, the integrin alpha IIb, to around 50% of the levels observed compared to the travel control tested simultaneously. When analyzed by the IT‐specific NGS panel a missense variant was identified in *ITGA2B*. This variant, c.2417G>A, p.Ser806Asn, is novel within all databases but predicted benign and not well conserved at the site of variation. The variant occurs within the extracellular domain and the integrin alpha IIb heavy chain. Although not predicted to, the reduction in cell surface CD41 is indicative of the possibility that the variant in *ITGA2B* affects either protein levels or cellular localization potentially leading to the observed platelet‐based bleeding phenotype. This is the only occurrence of a genotype‐phenotype correlation in all patients analyzed by the IT‐specific NGS panel. Although three variants were identified in *ITGA2B* and one variant was identified in *GP1BA*, none of the patients, with the exception of 61, were observed to have a reduction in the corresponding cell surface receptor levels.

Interestingly a reduction in cell surface expression of CD42b, encoded by *GP1BA*, was noted in patient 50, who harbors a potentially deleterious large deletion of *GP1BB* that spans two previously reported disease‐causing variants.^23^, ^24^ Although not occurring in the encoded gene, the variant, due to the detrimental effect of a frameshift causing deletion, may have propensity to disrupt the stability of the receptor complex leading to a reduction in cell surface expression.

As with variants determined by WES analysis, the variants observed following the application of the IT‐specific NGS panel require further conformational work to be determined disease causing. Further work would focus around this point mainly, utilizing many of the biomarkers of disease attributed to variants in certain genes and recruiting related affected family members of previously analyzed patients. This will strengthen any initial genetic variants that may be indicative of disease through segregation analysis but it also has the propensity to spread disease awareness of an under recognized and under‐diagnosed genetic disorder.

A possible lack of genotype–phenotype correlation shown in patients harboring variants in *ITGA2B*,* GP1BA*, and *MYH9* in particular is an interesting observation, however, further work would be needed to validate this. The possibility that these variants are disease causing rests on the functional confirmation of the effect of variation. However, if causative, the patients represent a unique subset of each individual disease that does not share the typical phenotypic presentation of previous cases. The likelihood that patients exist without the secondary symptoms and qualitative defects in platelet function attributed to these disorders is therefore relatively high.

Seven patients in total were observed without any variants in genes of the IT‐specific panel. The sequencing panel employed did not look at Copy Number Variations (CNVs) which could be present in the remaining patients studied. Due to the absence of variants within the panel of 30 genes, there is a high chance that the genetic etiology of disease is due to variants in novel genes not previously implicated in IT. Analysis of these patients in particular may progress our current knowledge of IT through the determination of novel causative genes.^25^

## Relationship Disclosure

All authors have no declarations of interest to report.

## Author Contributions

A significant portion of the findings of this study have originated from the thesis of Ben Johnson.^25^ Ben Johnson and Neil Morgan designed the research. Ben Johnson, Rachel Doak, Gillian Lowe, Yvonne Wallis, and Neil Morgan performed the research and analyzed data. David Allsup, Emma Astwood, Gillian Evans, Charlotte Grimley, Beki James, Bethan Myers, Simone Stokley, Jecko Thachil, Jonathan Wilde, Mike Williams, Mike Makris, Gillian Lowe, and Martina Daly provided patient samples and clinical data. Neil Morgan and Gill Lowe undertook the research governance of the study and coordinated the GAPP study. Ben Johnson and Neil Morgan wrote the paper and all authors critically reviewed and edited the paper.
